# The Effect of Copper And Zinc Nanoparticles on the Growth Parameters, Contents of Ascorbic Acid, and Qualitative Composition of Amino Acids and Acylcarnitines in *Pistia stratiotes* L. (Araceae)

**DOI:** 10.1186/s11671-016-1422-9

**Published:** 2016-04-23

**Authors:** Olga Olkhovych, Mykola Volkogon, Nataliya Taran, Lyudmyla Batsmanova, Inna Kravchenko

**Affiliations:** Educational and Scientific Centre “Institute of Biology”, Taras Shevchenko National University of Kyiv, 64, Volodymyrska str., Kyiv,, 01601 Ukraine

**Keywords:** *Pistia stratiotes*, Copper, Zinc, Nanoparticles, Amino acids, Acylcarnitine, Ascorbic acid, 82.70.Dd, 82.39.-k

## Abstract

The paper covers the research of copper and zinc nanoparticle effect on the content of ascorbic acid, and quantitative and qualitative composition of amino acids and acylcarnitines in *Pistia stratiotes* L. plants. Plant exposition to copper nanoparticles led to the decrease in (1) the amount of ascorbic acid, (2) the total content of amino acids (by 25 %), and (3) the amount of all studied amino acids except for the glycine amino acid. At this, the amount of 5-oxoproline, arginine, leucine, ornithine, phenylalanine, proline, serine, and tyrosine was two times lower than in control plants. The reduction of the contents of 8 out of 12 investigated acylcarnitines (namely C0, C2, C3, C5, C6, C8, C16, C18:1) was observed in plants under the influence of copper nanoparticles. The result of plants incubation with zinc nanoparticles was the decrease in (1) the amount of ascorbic acid, (2) the total content of amino acids (by 15 %), (3) the content of leucine, methionine, phenylalanine, proline, and tyrosine (more than twice), and (4) the content of 10 acylcarnitines (C0, C2, C3, C4, C5, C10, C16, C18, C18:1, C18:2). The observed reduction in amino acid contents may negatively affect plants adaptive reactions associated with de novo synthesis of stress proteins. At the same time, the decrease in the content of acylcarnitines, responsible for fatty acid transportation, may lead to the changes in the activity and direction of lipid metabolism in plants and reduce plant’s ability to use free fatty acids as the oxidation substrate for cell reparation.

## Background

Aquatic plants are successfully used around the world for remediation of the excessively transformed aqua systems. Pistia plants are known for their reclamation abilities through the accumulation of different water contaminants including heavy metals and are one of the main plant objects used for phytoremediation [11, 28]. This surface macrophyte is resistant to high concentrations of metals, possessing high growth and reproduction rates, both in artificial and natural aquatic ecosystems [10]. However, the effect of nanoparticles on its physiological and ecological characteristics has not been thoroughly studied yet and requires further investigation.

The increasing use of metal nanoparticles emphasizes the need in study of their toxicity and search for the potential plants that mitigate this environmental problem. It is known that metal nanoparticles cause changes in cell metabolism, changing the intensity of biochemical reactions that have a huge effect on plant’s resistance to various unfavorable conditions [4]. Copper and zinc nanoparticles were shown to be among the most toxic metal nanoparticles and metal oxides [8]. It was found that the presence of nanoparticles in aqueous solution leads to the formation of reactive oxygen species, primarily hydroxyl radicals [33], which increases their toxicity.

The aim of our study was to determine the influence of copper and zinc nanoparticles (Cu NPs and Zn NPs, respectively) on the metabolism of *Pistia stratiotes* in terms of its possible use for phytoremediation of industrial waters contaminated with metal nanoparticles.

## Methods

The studies were performed on the water macrophyte *P. stratiotes* L., which belongs to the *Araceae* family.

*P. stratiotes* plants were grown in aquatic complex of ESC “Institute of Biology” in 40–60-l aquariums with tap water under the optimal growth conditions: 6000-lx illumination, 18–22 °C water temperature, pH 5.8.

Zinc and copper were used in the form of metal nanoparticle solutions, contributed by the Department of the Technology of Construction Materials and Material Science of NULES (Ukraine). Colloidal solutions were obtained by dispersing copper and zinc granules with electric impulses with an amplitude of 100–2000 A in water [15]. The maximum size of nanoparticles has not exceeded 100 nm. The concentrations of metal nanoparticles in the stock solutions were 75 mg/l for Cu and 89 mg/l for Zn. Working solutions of metal NPs were obtained by the dilution of stock solutions with the water at 1:100 ratio.

The plants were grown in 0.3-l tanks of tap water with the corresponding solutions of metal NPs for 3 days under controlled growth conditions: illumination—5000 lx, temperature—19–27 °C, and 10-h light period.

The effect of studied NPs on *morphometric growth parameters* of pleistophyte was determined in the model experiment measuring plant weight and number of plants, leaves, and roots per plant and newly formed plants. Measurements were performed at the beginning of the experiment, as well as on the 7th and 14th day of experiment.

The contents of ascorbic acid, total protein, amino acids, and acylcarnitines were determined in the dry matter of *P. stratiotes* plants.

*Determination of ascorbic acid* was performed according to Muri [14].

*Determination of total protein* was performed using biuret method. A sample of air-dry plants (0.1 g) was homogenized with 0.5 g of glass powder and 0.5 g of anhydrous Na_2_(SO_4_). The homogenate was transferred to a glass column with a filter and filtered after adding to it 3 ml of acetone.

Remaining dry material from filter was transferred to a test tube, washed with 4 ml of 2.5 % trichloroacetic acid, and centrifuged for 5 min at 5000 *g*. After removal of supernatant, the procedure was repeated. Then, the similar procedure was performed using 5 ml of distilled water. On the next step, 5 ml of 0.05 M NaOH was added to the test tube with dry material and centrifuged. After centrifugation, 3 ml of top fractions were mixed with 0.5 ml of biuret reagent [9] and used for the measurement of optical density (*λ* = 550 nm) on “Shimadzu UV-1800” spectrophotometer.

*Determination of amino acids and acylcarnitines* was performed using tandem mass spectrometry [20] with the AB Sciex 2000 Autosampler Ultimate 3000 (Dionex) mass spectrometer. In the analysis, the 3-mm-diameter disks were used. During samples preparation for each probe (20-ml extract, used for the protein determination), the 200 ml of internal standard (the mixture of deuterium-labeled amino acids and acylcarnitines with known concentrations) was added. After incubation with an internal standard, samples were dried before the subsequent derivatization using 3 M butanol/HCl solution. After drying out samples were dissolved in the reconstitutional buffer and loaded to the Ultimate 3000 sampler for analysis. Quantitative study of mixture of amino acids and acylcarnitines was performed by comparing chromatograms of standard and experimental mixtures of amino acids and acylcarnitines [24].

The experiment was conducted with at minimum three biological and analytical replications. The data analysis was performed using Microsoft Office Excel, Student *t* test with the significance level *p* ≤ 0.05.

## Results and Discussion

### Growth Parameters of *P. stratiotes* Plants

Our results indicate an ambiguous impact of the studied copper and zinc nanoparticles on growth of *P. stratiotes*, possessing simultaneously stimulating and inhibitory action (Table 1).

**Table 1:** The effect of Cu and Zn nanoparticles at their individual and combined application on growth parameters of *P. stratiotes*

Variant	Plant weight, g	Number of leaves per plant, pcs.	Number of roots per plant, pcs.	Number of plants, pcs.
1st day of experiment				
Check	0.183 ± 0.011	9 ± 0.6	21 ± 0.3	3
Сu + Zn NPs	0.158 ± 0.012	10 ± 0.3	23 ± 0.4	3
Zn NPs	0.117 ± 0.012	8 ± 0.1	20 ± 0.3	3
Сu NPs	0.150 ± 0.009	9 ± 0.3	21 ± 0.2	3
7th day of experiment				
Check	0.252 ± 0.004	12 ± 0.4	28 ± 0.6	4
Сu + Zn NPs	0.224 ± 0.021	12 ± 0.1	27 ± 0.3	4
Zn NPs	0.167 ± 0.018	11 ± 0.2	26 ± 0.5	3
Сu NPs	0.172 ± 0.019	12 ± 0.1	23 ± 0.2	4
14th day of experiment				
Check	0.290 ± 0.008	14 ± 0.2	37 ± 0.5	5
Сu + Zn NPs	0.268 ± 0.015	19 ± 0.5	32 ± 0.2	4
Zn NPs	0.219 ± 0.013	13 ± 0.2	33 ± 0.4	3
Сu NPs	0.191 ± 0.024	13 ± 0.3	25 ± 0.2	4

During the first week of growth of *P. stratiotes* plants, no discoloration or growth-inhibiting effects were observed in the studied variants which resulted in the increase of plant weight, as well as the number of roots and leaves per plant on the 7th day of the experiment.

**Fig. 1:**
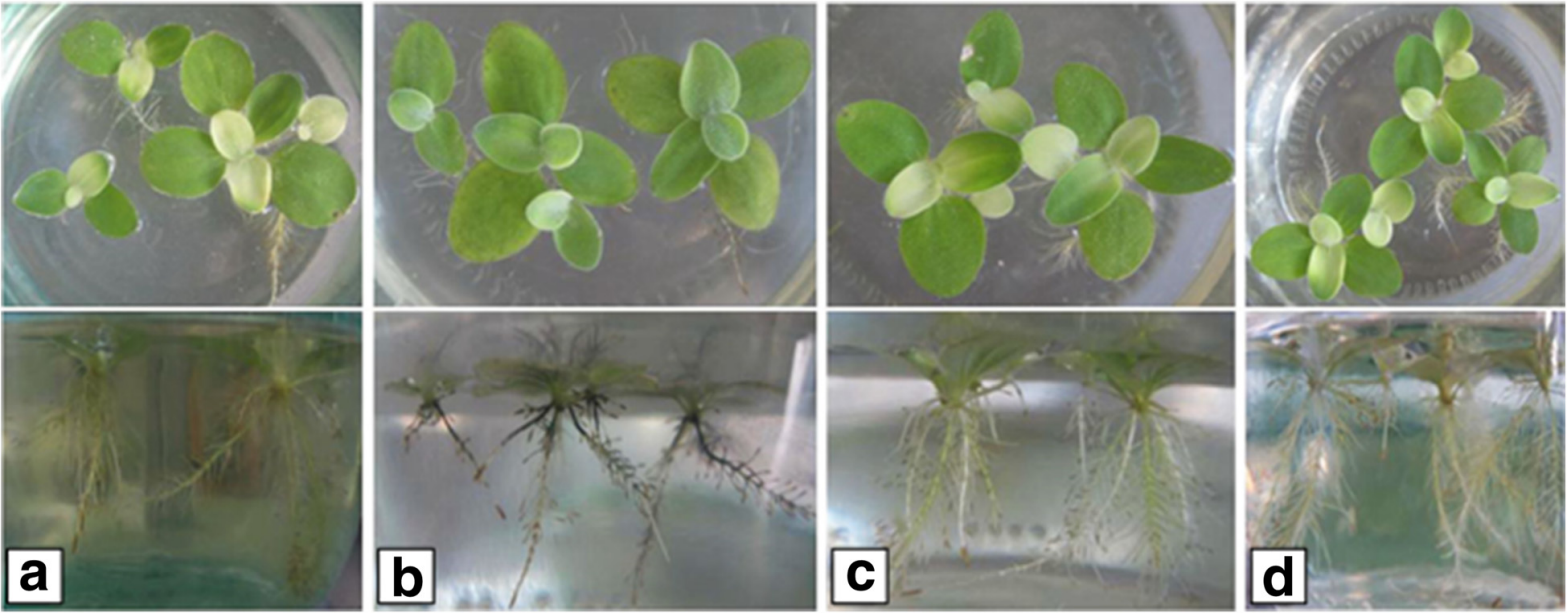
Morphological changes of leaves and roots of *Pistia stratiotes* L. plants under the influence of metal nanoparticles on the 14th day of growth: **a** check, **b** copper nanoparticles, **c** zinc nanoparticles, **d** binary composition of copper and zinc nanoparticles

The increase of plants weight was observed in all experimental variants on the 14th day of the experiment, followed by the increase in the number of leaves and roots per one plant. However, leaves discoloration from dark green to light green color was observed in variants with of Zn NPs and binary composition of Cu and Zn NPs, which may indicate the breakdown of chlorophyll, especially chlorophyll *a*. At the same time, the intense growth of lateral roost in these variants was observed (Fig. 1c, d).

Plants grown in the presence of Cu NPs remained green but with the signs of turgor loss in mesophilic cells. Besides that, the blackening and perishing of existing roots followed by inhibition of new growth was observed (Fig. 1b).

The effect of binary composition of metal nanoparticles on *P. stratiotes* plants indicated the synergic action of Cu and Zn NPs. Thus, plants from this variant had light green color and subnormal turgor of leave cells and the highest leaf growth, but low increase in root number.

Overall, the influence of Zn NPs and their binary composition with Cu NPs on morphological parameters of *P. stratiotes* L. was more obvious compared to the effect of copper nanoparticles.

### Protein and Amino Acid Contents

The exchange of proteins and lipids plays an important role in plant metabolism under the stress of any nature. Protein compounds, such as enzyme complexes, which quickly respond to the changing environmental conditions, and stress proteins, synthesized de novo, both ensure plant’s resistance to stress.

**Fig. 2:**
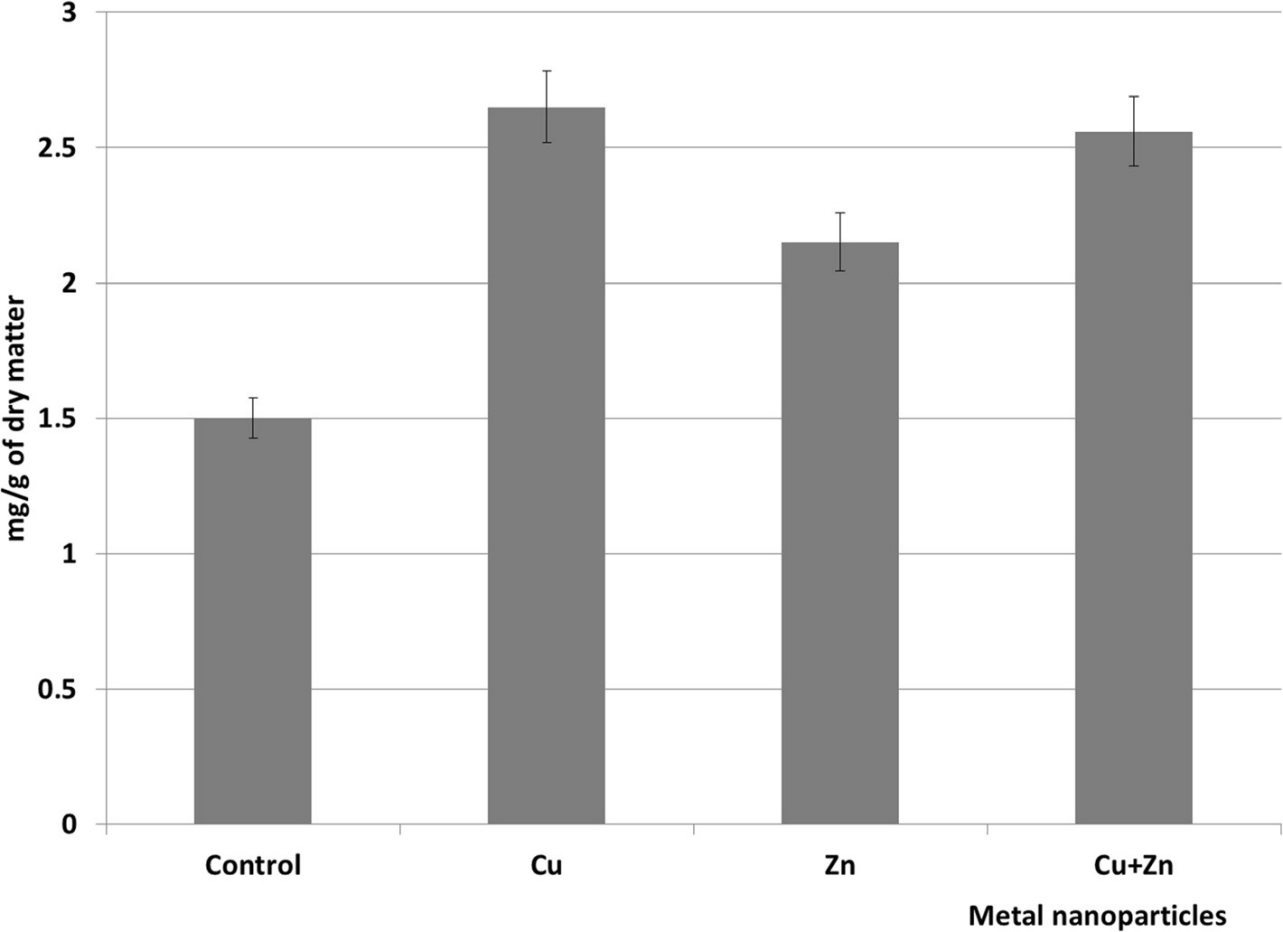
The protein content of *P. stratiotes* under the influence of copper and zinc nanoparticles (Control, Cu—Cu nanoparticles, Zn—Zn nanoparticles, Cu + Zn—binary composition of Cu and Zn nanoparticles)

The observed increase of protein content in plants exposed to metal NPs (copper, zinc, and their binary composition) have proved our assumption that protein content in *P. stratiotes* in experimental variants will differ from the norm (Fig. 2).

**Fig. 3:**
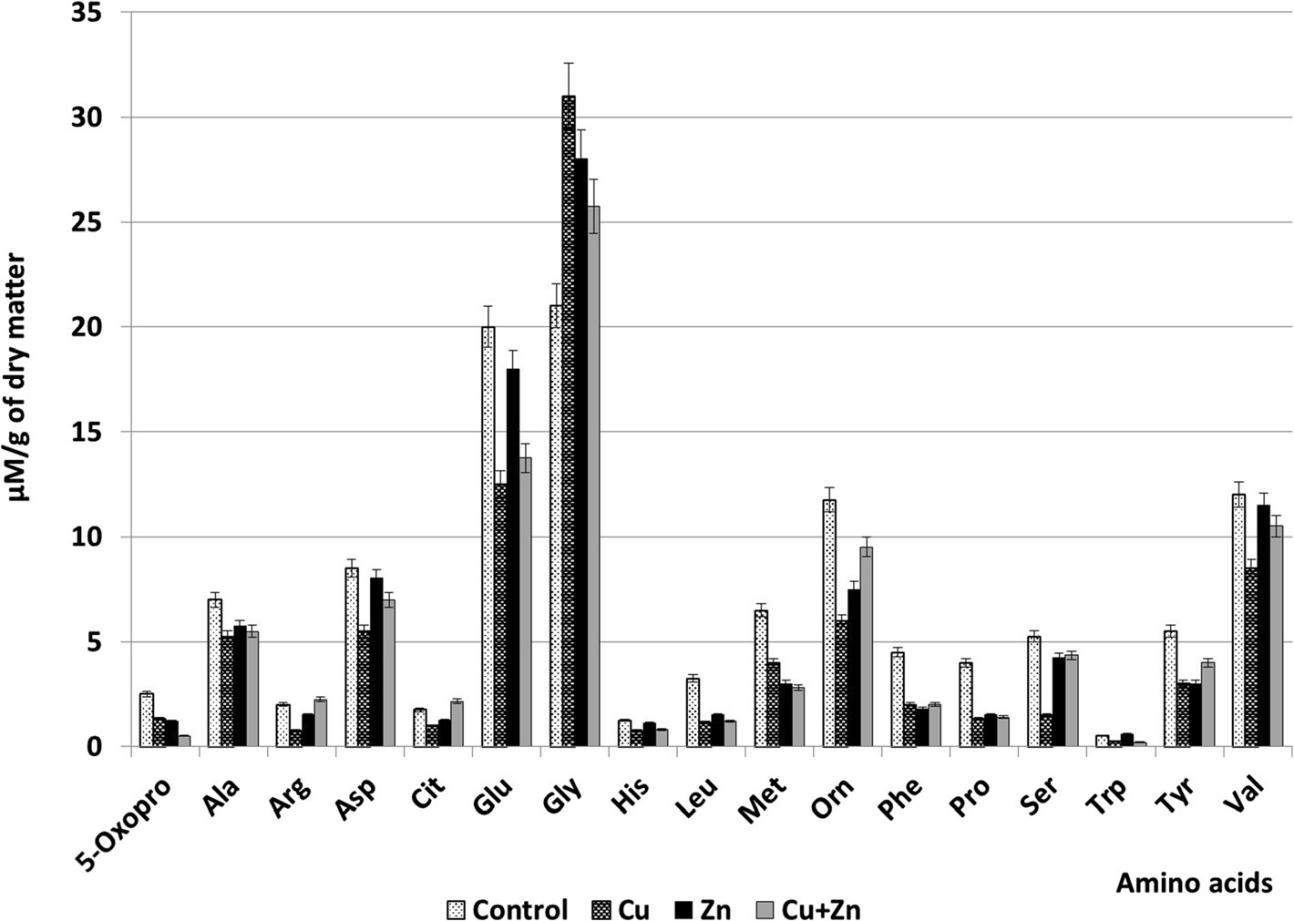
The content of amino acids in *P. stratiotes* plants exposed to the action of copper and zinc nanoparticles and their binary composition

Besides differences in total protein content, variations in the amount and ratio of amino acids were also revealed. Thus, 17 amino acids from *P. * plants (both in test and check plants) were identified and analyzed (5Oxo-Pro—5-oxoproline, Ala—alanine, Arg—arginine, Asp—aspartic acid, Cit—citrulline, Glu—glutamic acid, Gly—glycine, His—histidine, Leu—leucine, Met—methionine, Orn—ornithine, Phe—phenylalanine, Pro—proline, Ser—serine, Trp—tryptophan, Tyr—tyrosine, Val—valine). The obtained results are shown in Fig. 3.

Comparative analysis of the effect of metal NPs has shown that the total content of amino acids in the studied plants of *P. stratiotes* was 88.2 μmol/g in plants under the influence of copper NPs and 101.5 μmol/g under the influence of zinc NPs, thus indicating a stronger negative effect of copper comparing to zinc NPs.

The decrease in all studied amino acids with the exception of Gly was observed in *P. stratiotes* plants treated with Cu NPs. The content of some amino acids (5Oxo-Pro, Arg, Leu, Orn, Phe, Pro, Ser, Tyr) decreased by more than two times. In contrast, the content of amino acids in plants treated with Zn NPs did decreased so dramatically as under the influence of copper nanoparticles. The content of only five amino acids—Leu, Met, Phe, Pro, and Tyr—decreased by more than two times. The content of four amino acids (Leu, Phe, Tyr, and Pro) was the same in the presence of Zn NPs as for Cu NPs. Reduction of these amino acid content, especially proline, affects plant’s ability to form adaptive responses as proline amino acid is known as acceptor of radicals, stabilizer of macromolecules [12], and a chelating compound [34]. Different studies have shown that many plants accumulate proline amino acid under stress conditions (water deficit, low and high temperatures, high salinity) [2, 3, 21]. Moreover, in studies of *Chlamydomonas reinhardtii* algae, the direct relation of proline amino acid content to plant’s resistance to metals was revealed [31].

Histidine is known to be produced by plants capable of accumulating zinc and other metals [27]. The reduction of His amino acid content in *P. stratiotes* plants under the action of both Cu and Zn NPs may indicate low ability of plants to accumulate these metals. Nevertheless, it may also testify on the use of different complex mechanisms by *P. stratiotes* plants, similar to the Zn-histidine, but with another amino acid. Our research revealed the increase in the content of glycine amino acid under the action of both studied nanoparticles. Glycine and glutamic acid take part in the synthesis of glutathione and other phytochelating agents known for binding heavy metals [29]. These findings suppose the existence of specific plant’s reaction on the presence of metal nanoparticles but might require further investigation on its specific mechanism.

Another important amino acid in the synthesis of polyamines that acts as the signaling molecule and an antioxidant agent during defensive reactions in plants is arginine. Under the action of Cu NPs, its contents decreased two times, while under the action of Zn NPs, it has remained at a control level, thus indicating an inappropriateness of its involvement in *P. stratiotes* metabolism under the action of metal NPs. Our results were confirmed by the data on the synthesis of different metabolites, including amino acids, at plant’s interaction with heavy metals [34].

Increase of amino acid content in plants is aimed to support homeostasis, vital for plants, but the rise of single amino acid content out of 17 under the action of the studied nanoparticles contradicts the involvement of adaptive responses in *P. stratiotes* plants.

Joint action of copper and zinc nanoparticles in binary composition resulted in decrease in content of Asp, Glu, His, Val amino acids less comparing to the action of using single Cu NPs, but more if compared to the effect of Zn NPs. The content of Orn, Ser, and Tyr amino acids was higher than in plants influenced by the copper and zinc nanoparticles separately.

### Acylcarnitine Content

Rapid changes in membrane lipid composition are essential for plant’s resistance to the unfavorable factors, including heavy metals, ensuring penetration through the membranes of specific substances (fatty acids, in particular). Transport of fatty acids through the plant cell organelles’ membranes is actively supported by acylcarnitines [22]. Acylcarnitines are derivatives of long-chain fatty acids and are required for their transportation into the mitochondria for β-oxidation [19]. They cannot be considered as products of enzyme system of carnitine transport, but indirectly they point to altered mitochondrial metabolism [25], since they both are involved into mitochondrial β-oxidation. The distortion of their endogenous pool is associated with mitochondrial dysfunction [22].

There is evidence of carnitine and acylcarnitine presence in plant cells and their role in the plant metabolism, including transportation of acyl-CoA molecules through the mitochondrial membrane [17, 22, 26]. The discovery of carnitine (acetyl, octanoyl, palmitoyl) transferase activity in the mitochondria of pea and bean sprouts, as well as the identification of acylcarnitine quantitative composition in some plant species (*Arabidopsis thaliana*, canola, flax, tobacco) [5], has proven the carnitine role in the metabolism of lipids in plants. It is known that carnitine contents in plant tissues are significantly lower than those in animal tissues and acylcarnitines constitute less than 2 % of total pool of carnitine. It is also known that some fatty acids do not require cytosolic carnitine and CoA for their transportation to the mitochondria [22, 23]. Despite the fact that acylcarnitines play a minor role in lipid metabolism of plants [5] compared to animals, their importance for plants resistance to metals can be significant.

**Fig. 4:**
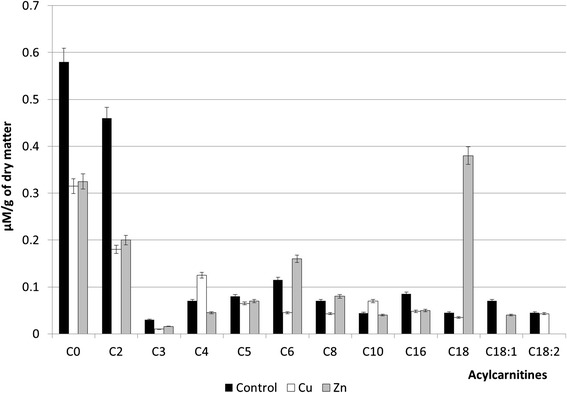
Acylcarnitine content in *P. stratiotes* plants exposed to the action of copper and zinc nanoparticles

We have determined the contents of 12 acylcarnitines in *P. stratiotes* plants in normal conditions and under the action of copper and zinc nanoparticles, as follows: C0—free carnitine, C2—acetylcarnitine, C3—propionyl carnitine, C4—butyryl carnitine, C5—isovaleryl carnitine, C6—hexanoyl carnitine, C8—octanoylcarnitine, C10—decanoyl carnitine, C16—palmitoyl carnitine, C18—stearyl carnitine, C18:1—oleyl carnitine, C18:2—linoleyl carnitine (Fig. 4).

It was established that the contents of two acylcarnitines, namely C4 and C10, in *P. stratiotes* plants increased in the variants with Cu NPs, while the contents of C18 and C18:2 acylcarnitines remained at control level. The content of other acylcarnitines (C0, C2, C3, C5, C6, C8, C18) decreased significantly, while C18:1 acylcarnitine was not determined.

Taking the abovementioned into account, it can be assumed that the impact of Cu NPs might interfered in the transfer of specific fatty acids through the mitochondria membrane and, consequently, had reduced respiration level and energy metabolism in cells.

Study of zinc nanoparticle influence on *P. stratiotes* plants revealed decrease in nine acylcarnitine contents and significant increase of the C6 and C8 acylcarnitine contents, while C18:2 was not identified. Linoleyl carnitine (C18:2) appeared to be the most sensitive to the effect of Zn NPs.

It should be noted that under the action of copper and zinc nanoparticles, the content of individual acylcarnitines was changed in different ways, but in both cases, there were changes in lipid metabolism aimed at the maintenance of plant homeostasis under the action of metals.

Literature data show the role of carnitine in the formation of *A. thaliana* resistance to the salinity and oxidative stress conditions through the increase of seedling growth rates and restoration [6]. Based on these findings and thorough analysis of the obtained results, we suppose the same role of carnitine in aquatic plants under the adverse influence of metal nanoparticles.

The decrease of C0 contents under the action of both metal nanoparticles was observed. Free carnitine (C0) or l-3-hydroxy-4-*N*-trimethylaminobutyrate is a quaternary ammonium compound, which plays a major role in fatty acid transport in living cells [16]. Our data indicate that metal nanoparticles affect the conversion process of carnitine to acylcarnitines resulting, respectively, in the reduction of their contents and, in some cases, their vanishing.

Normal ratio of acylcarnitines to carnitine contents in plant cells is 0.25. The ratio of 0.4 and above is considered abnormal and indicates on the disorders in lipid metabolism [34]. In our studies, the distortion of the C0 and C2 ratio was observed under the action of nanoparticle solutions of both metals, while the ratio among other acylcarnitines and carnitine remained within the normal limits. Thus, acetylcarnitine appeared to be the most sensitive to the damaging effect of nanoparticles of studied metals.

### Ascorbic Acid

The formation of free radicals in plants is stimulated under any stress. The antioxidant system of plants provides various mechanisms to compete against the oxidative stress and involves both low molecular weight antioxidants (pigments, ascorbic acid, flavonoids, etc.) and specific enzymes. Most of current studies are focused on the usage of low molecular weight antioxidant content as biomarkers of physiological state of plants, since their content increases under stress conditions [1].

Ascorbic acid is of particular interest among the natural antioxidants. It is able to oxidize to dehydroascorbic acid, and it is believed that these two molecules form an effective redox system characterized with a high vitamin activity [32]. Reduced and oxidized ascorbic acid is present in plants in free state. Ascorbic acid serves as a substrate for ascorbate peroxidase, which is a key enzyme of ascorbate cycle. Maintaining a high ratio of the reduced form of ascorbic acid over the oxidized is required for the protection of plant cells against ROS. This ratio, maintained by monodehydroascorbate reductase and dehydroascorbate reductase, takes part in the creation of physical barrier from heavy metal entry to plant cells [30]. The synthesis of ascorbic acid in plants depends on many factors, including heavy metal content [18]. Ascorbic acid plays an important role in photosynthesis, especially in stabilizing the photosynthetic apparatus, thus enhancing photochemical activity of plants.

**Fig. 5:**
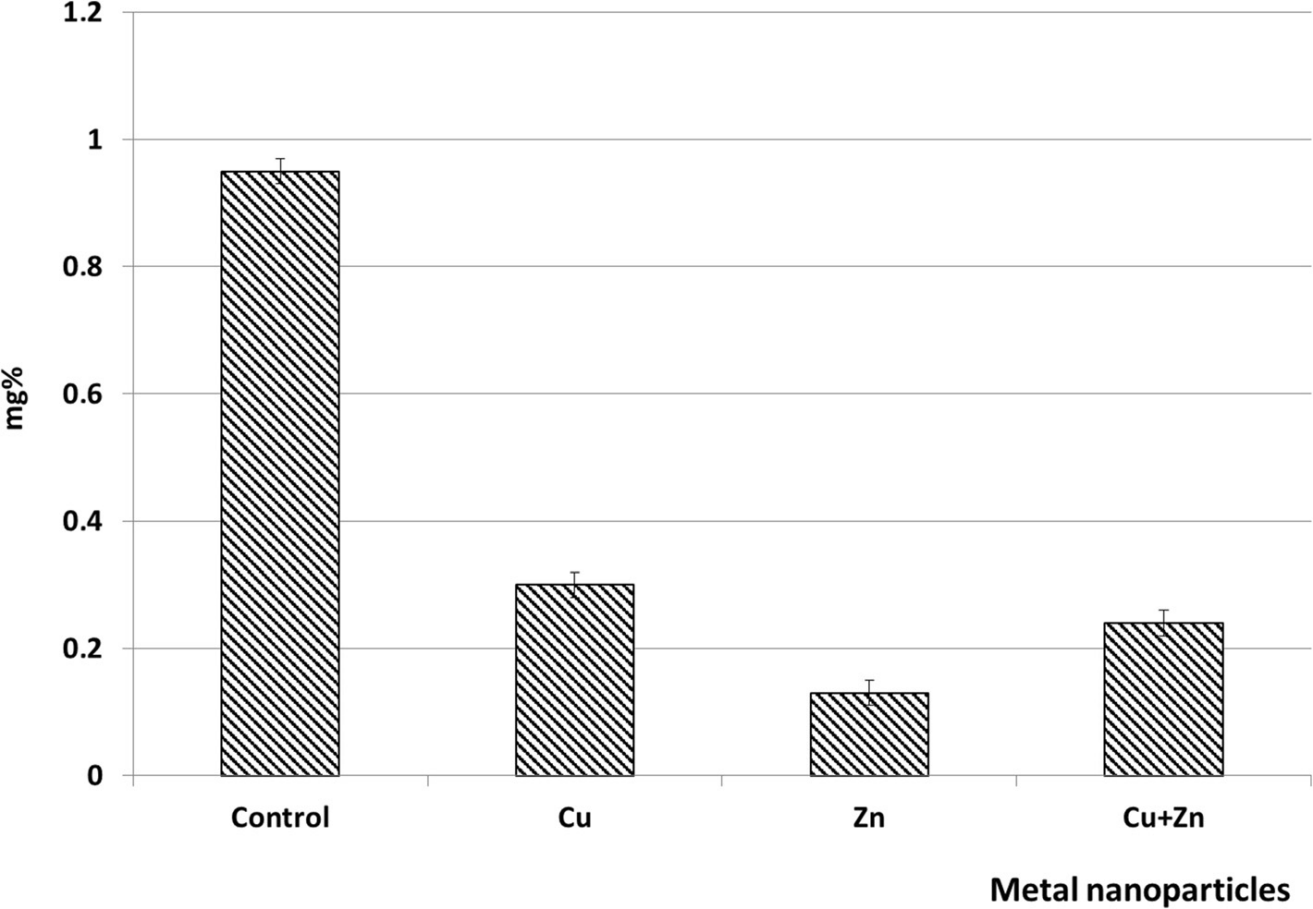
The content of ascorbic acid in *P. stratiotes* plants exposed to the action of copper and zinc nanoparticles and their binary composition

Our findings indicated a decrease in ascorbic acid content under the action of studied biogenic metal nanoparticles (Fig. 5).

Under the influence of Cu NPs, the content of ascorbic acid is reduced by 63 %. The 85 % decrease of ascorbic acid content was observed in variants with Zn NPs, while their joint presence resulted in 70 % reduction of ascorbic acid content. This may be caused by inhibitory effect of metal ions on enzyme activity of ascorbic acid metabolism and reduction of its pool due to the ROS neutralization and its use for plant growth and development, which was confirmed by studies on other plants [7, 35]. Ascorbic acid plays an important role in plant morphogenesis and indicates plant’s ability to respond to the environmental changes [35]. The long-term effects of copper and zinc nanoparticles on *P. stratiotes* plants may consist in irreversible destructive changes that in turn may have happened due to the exhaustion of ascorbic acid resources in plants. Nevertheless, there is still a possibility of plant response aimed to neutralize produced ROS formed at increase of metal ions in the cytoplasm [13].

Literature data indicate that copper and zinc have a synergistic effect when applied together. Namely, the toxicity of zinc and copper was eight times higher than the toxicity of each of these metals taken separately [29]. Our data had also indicated a synergistic effect of Cu and Zn NPs on *P. stratiotes* plants, but less intense. However, it should be noted that it is much harder to clean composite wastewaters with water plants compared to a single or a low-compound contaminated waters while in the case of highly toxic component contamination, this task become impossible. This should be considered when using water on enterprises, which should avoid unnecessary intermixing of toxic and low toxic wastes from various cycles, especially when containing nanoparticles of different metals, since the action of such compositions can be unpredictable for plants that can remove or neutralize pollutants from a contaminated water.

## Conclusions

The colloidal solutions of copper and zinc nanoparticles had a negative but not critical effect on the metabolism of *P. stratiotes* plants, thus indicating their possible use for phytoremediation of industrial waters contaminated with metal nanoparticles when proved to be able to adsorb the metal nanoparticles from solutions.

Our studies reveled reduced ascorbic acid content, increased total protein content, decreased by 25 % total amino acid contents, and reduced contents of all amino acids, except for glycine, as well as decreased contents of 8 out of 12 identified acylcarnitines (namely C0, C2, C3, C5, C6, C8, C16, C18:1) in *P. stratiotes* plants under the action of copper nanoparticles.

The same effects though in varying degrees were observed in *P. stratiotes* plants under the action of zinc nanoparticles: reduced ascorbic acid content, increased total protein content, decreased by 15 % total contents of amino acids, as well as decreased contents of 10 acylcarnitines (C0, C2, C3, C4, C5, C10, C16, C18, C18:1, C18:2).

The binary composition of copper and zinc nanoparticles had a synergistic effect on *P. stratiotes* plants that should be taken into account while using this plant for phytoremediation of wastewater containing nanoparticles of both metals.

The reduction of amino acid content under the action of metal nanoparticles negatively affects plant’s ability to form adaptive reactions associated with the synthesis of stress protein. The decrease of acylcarnitine content that besides other functions assist in transportation of fatty acids to the mitochondria may cause changes in the activity and orientation processes not only of the metabolism of lipids but also in energy metabolism associated with them.

## References

[1] Abramova EA, Ivanyschev VV (2012). Contents of photosynthetic pigments and ascorbic acid in vetch seedlings of under the influence of nickel chloride. Scientific letters. Life Sci Ser.

[2] Ashraf M, Harris PJC (2004). Potential biochemical indicators of salinity tolerance in plants. Plant Sci.

[3] Aspinall D, Paleg LG, Paleg LG, Aspinall D (1981). Proline accumulation: physiological aspects. The physiology and biochemistry of drought resistance in plants.

[4] Bhattacharya D, Gupta RK (2005). Nanotechnology and potential of microorganisms. Crit Rev Biotechnol.

[5] Bourdin B, Adenier H, Perrin Y (2007). Carnitine is associated with fatty acid metabolism in plants. Plant Physiol Biochem.

[6] Charrier A, Rippa SYA, Nguyen PJ, Renou JP, Perrin Y (2012). The effect of carnitine on Arabidopsis development and recovery in salt stress conditions. Planta.

[7] Chupakhina GN (1997) Ascorbic acid system in plants: monograph. Kaliningrad, KSU [*in Russian*]

[8] Crane M, Handy RD (2007). An assessment of regulatory testing strategies and methods for characterizing the ecotoxicological hazards of nanomaterials. Rep Defra Lond UK.

[9] Gornall AG, Bardawill CJ, David MM (1949). Determination of serum proteins by means of the biuret reaction. J Biol Chem.

[10] Henry-Silva GGI, Camargo AFM (2006). Composição química de macrófitas aquáticas flutuantes utilizadas no tratamento de efluentes de aqüicultura. Planta Daninha.

[11] Henry-Silva GGI, Camargo AFM (2006). Composição química de macrófitas aquáticas flutuantes utilizadas no tratamento de efluentes de aqüicultura. Planta Daninha.

[12] Karakaeva LS, Dokuchaeva YA, Mashkova AA (2013). Contents of ascorbic acid and heavy metals in the *Populus* L. plants from different zones around the Orenburg. Proc Orenburg State Agricult Univ.

[13] Koshkyn E.I. (2010) Physiology of agricultural crops resistance. Dropha, Moscow, 2010 [*in Russian*]

[14] Lobanov O, Ageev VV, Esaulko AN et al. (2010) Laboratory work book on food chemistry. Agrus, Stavropol [*in Russian*]

[15] Lopatko KH, Aftandiliants EH, Kalenska SM, Tonkha OL. Mother colloidal solution of metals. Patent for invention 38459 from 12.01.2009.

[16] Mansour FR, Wei W, Danielson ND (2013). Separation of carnitine and acylcarnitines in biological samples: a review. Biomed Chromatogr.

[17] Masterson C, Wood C (2000). Mitochondrial oxidation of fatty acids in higher plants. Physiol Plant.

[18] Matysik J, Alia B, Mohanty P (2002). Molecular mechanisms of quenching of reactive oxygen species by proline under stress in plants. Curr Sci.

[19] McGill MR, Li F, Sharpe MR, Williams CD, Curry SC, Ma X, Jaeschke H (2013). Circulating acylcarnitines as biomarkers of mitochondrial dysfunction after acetaminophen overdose in mice and humans. Arch Toxicol.

[20] Mikhaylova SV, Baydakova GV, Boukina AM, Boukina TM, Shechter OV, Ilina ES, Zakharova EY (2004). Combination of tandem mass spectrometry and lysosomal enzymes analysis—effective tool for selective screening for IEM in neurological clinic. J Inherit Metab Dis.

[21] Naidu BP, Paleg LG, Aspinall D, Jennings AC, Jones G (1991). Amino acid and glycine-betaine accumulation in cold stressed seedlings. Phytochemistry.

[22] Nguyen PJ, Rippa S, Rossez Y, Perrin Y. Acylcarnitines participate in developmental processes associated to lipid metabolism in plants. Planta. 2016. DOI 10.1007/s00425-016-2465-y. 10.1007/s00425-016-2465-y 26748916

[23] O’Donnel JM, Alpert NM, White LT, Lewandowski ED (2002). Coupling of mitochondrial fatty acid uptake to oxidative flux in the intact heart. Biophys J.

[24] Ovchynnikova YA (1974) New methods of analysis of amino acids, peptides and proteins. Мyr, Moscow [in Russian].

[25] Reuter SE, Evans AM (2012). Carnitine and acylcarnitines: pharmacokinetic. Pharmacol Clin Aspects.

[26] Rippa S, Zhao Y, Merlier F, Charrier A, Perrin Y (2012). The carnitine biosynthetic pathway in Arabidopsis thaliana shares similar features with the pathway of mammals and fungi. Plant Physiol Biochem.

[27] Salt DE, Prince RC, Baker AJM, Raskin I, Pickering IJ (1999). Zinc ligands in the metal hyperaccumulator Thlaspi caerulescens as determined using X-ray absorption spectroscopy. Environ Sci Tech.

[28] Semenchenko VP (2004) The principles and systems of bioindication of flowing waters. National Academy of Sciences of Belarus. Orekh, Minsk [*in Russian*]

[29] Sharma SS, Dietz KJ (2006). The significance of amino acids and amino acid-derived molecules in plant responses and adaptation to heavy metal stress. J Exp Bot.

[30] Sinha S, Basant A, Malik A (2009). Multivariate modeling of chromium-induced oxidative stress and biochemical changes in plants of Pistia stratiotes L. Ecotoxicology.

[31] Siripornadulsil S, Traina S, Verma DPS, Sayre RT (2002). Molecular mechanisms of proline-mediated tolerance to toxic heavy metals in transgenic microalgae. Plant Cell.

[32] Smirnova OV, Efimova IV, Opeyda IA (2014). Features of radical chain oxidation of ascorbic acid in an aprotic environment. Scientific letters of DonNTU. Ser Chem Chem Technol.

[33] Thurn KT, Brown EMB, Wu A, Vogt S, Lai B, Maser J, Paunesku T, Woloschak GE (2007). Nanoparticles for applications in cellular imaging. Nanoscale Res Lett.

[34] Vesely T, Neuberg M, Trakal L, Szakova J, Tlustoa P (2012). Water lettuce *Pistia stratiotes* L. response to lead toxicity. Water Air Soil Pollut.

[35] Zelenchukova NS, Ivanytskyy AE, Tayupova RR (2015). Features of growth and development of *Eruca sativa* under the polyethylene film low emission cover based on copper and silver compounds. Bull TSPU.

